# Tuning of resistive memory switching in electropolymerized metallopolymeric films†
†Electronic supplementary information (ESI) available: CVs of the poly-**1** ^4+^/Pt film at different scan rates, FTIR spectra of **1**(PF_6_)_4_ and poly-**1** ^4+^, AFM images of poly-**1** ^4+^/ITO and poly-**2** ^2+^/ITO films and XPS spectrum of poly-**1** ^4+^/ITO film, oxidative electropolymerization of **2**(PF_6_)_2_, HOMO and LUMO plots of the basic monoruthenium-tetraphenylbenzidine structure of poly-**2** ^2+^, full list of authors of ref. 22, Cartesian coordinates of the DFT-optimized structure, NMR and mass spectra of new compounds, and the CIF file for **1**(PF_6_)_4_. CCDC 1023945. For ESI and crystallographic data in CIF or other electronic format see DOI: 10.1039/c4sc03345k



**DOI:** 10.1039/c4sc03345k

**Published:** 2014-11-24

**Authors:** Bin-Bin Cui, Zupan Mao, Yuxia Chen, Yu-Wu Zhong, Gui Yu, Chuanlang Zhan, Jiannian Yao

**Affiliations:** a Beijing National Laboratory for Molecular Science , CAS Key Laboratory of Photochemistry , Institute of Chemistry , Chinese Academy of Sciences , Beijing 100190 , China . Email: zhongyuwu@iccas.ac.cn ; Email: clzhan@iccas.ac.cn ; Email: jnyao@iccas.ac.cn ; Fax: +86 010 62559373 ; Tel: +86 010 62652950; b Key Laboratory of Organic Solids , Institute of Chemistry , Chinese Academy of Sciences , Beijing 100190 , China . Email: yugui@iccas.ac.cn

## Abstract

A diruthenium complex capped with two triphenylamine units was polymerized by electrochemical oxidation to afford metallopolymeric films with alternating diruthenium and tetraphenylbenzidine structures. The obtained thin films feature rich redox processes associated with the reduction of the bridging ligands (tetra(pyrid-2-yl)pyrazine) and the oxidation of the tetraphenylbenzidine and diruthenium segments. The sandwiched ITO/polymer film/Al electrical devices show excellent resistive memory switching with a low operational voltage, large ON/OFF current ratio (100–1000), good stability (500 cycles tested), and long retention time. In stark contrast, devices with polymeric films of a related monoruthenium complex show poor memory performance. The mechanism of the field-induced conductivity of the diruthenium polymer film is rationalized by the formation of a charge transfer state, as supported by DFT calculations.

## Abstract 2

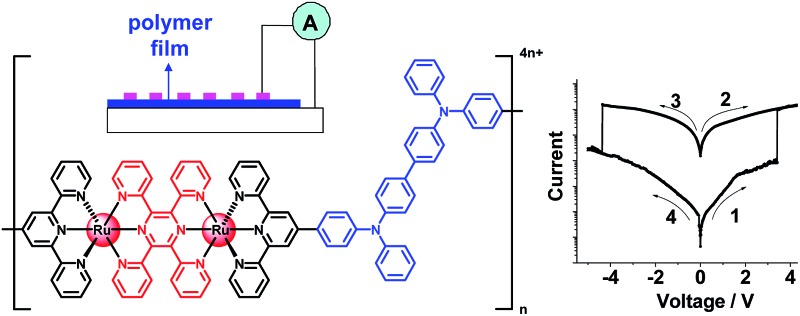
Sandwiched electrical devices of an electropolymerized diruthenium metallopolymeric film show excellent resistive memory switching.

## Introduction

Thin films of semiconducting organic and polymeric materials have received tremendous interest for a wide range of optical and electrical applications.^1^ Among them, resistive memory devices based on these films hold great promise for high-density data storage with a miniaturized device size.^2^ A resistive memory, or memristor, operates as an electrical switch between high and low conductivity states (multi-states are possible) and remembers its present resistance when the electric power supply is turned off.^2,3^ Organic and polymeric materials have a number of advantages for use as memory elements, such as structural tunability and diversity, good scalability, low cost, low power consumption, flexibility, multilevel storage, and large capacity.^2,4,5^

**Fig. 1:**
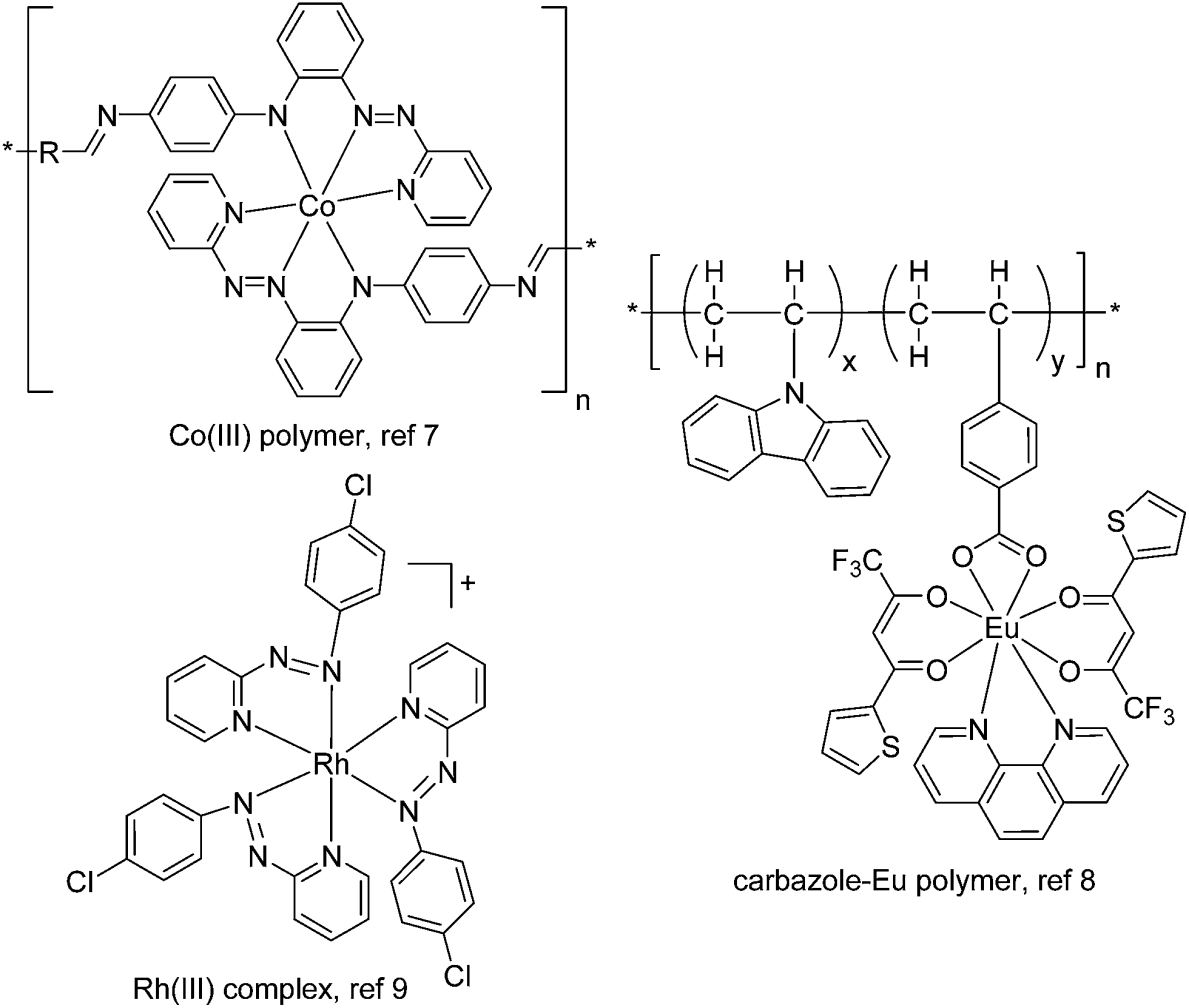
Representative known transition metal complexes or metallopolymers as active layers for resistive memory. The thin films of these materials were prepared by spin-coating.

The molecular design and film formation of the active materials are crucial to the performance of memory devices. Vacuum-deposited or solution-processed films of small organic molecules (often with multiple charge-trapping sites) have been reported to exhibit excellent memory behaviour with high ON/OFF current ratios and multilevel storage.^4^ Memory devices based on solution-processed polymeric films (often with donor–acceptor structural components) have shown promising switching performance.^5^ Recently, the incorporation of transition metal complexes into memory devices has also received much interest (Fig. 1), due to their well-defined and tunable redox properties.^6^ For instance, Higuchi has fabricated memristive devices using electrochemically active Co(iii) polymers.^7^ Poly-*N*-vinylcarbazoles with on-chain Eu(iii) or Ir(iii) complexes have been demonstrated to give bistable or ternary memory devices.^8^ Goswami reported an azo anion radical complex of Rh(iii) as an active layer for molecular memory switching devices.^9^ Despite these advances, materials with tailored electronic properties, in conjunction with a good film formation method, are still in urgent demand for developing high-performance memory devices.

Electropolymerization is a very convenient method for the formation of thin films, where the polymerization is electrochemically initiated and the polymers are deposited *in situ* on electrode surfaces to afford adhesive films.^10^ This procedure significantly shortens the experimental time and avoids the solubility issues often encountered with other methods. In addition, the equipment needed to carry out electropolymerization is much simpler and cheaper relative to vacuum deposition. Electropolymerized films of organic or organometallic monomers have been reported to show memory functions with optical outputs.^11^ However, both inputs and outputs in the form of electrical signals are preferred for practical data storage technologies. We present herein the first example of using a metal-containing electropolymerized thin film as the active layer for promising resistive memory devices.

## Results and discussion

**Scheme 1:**
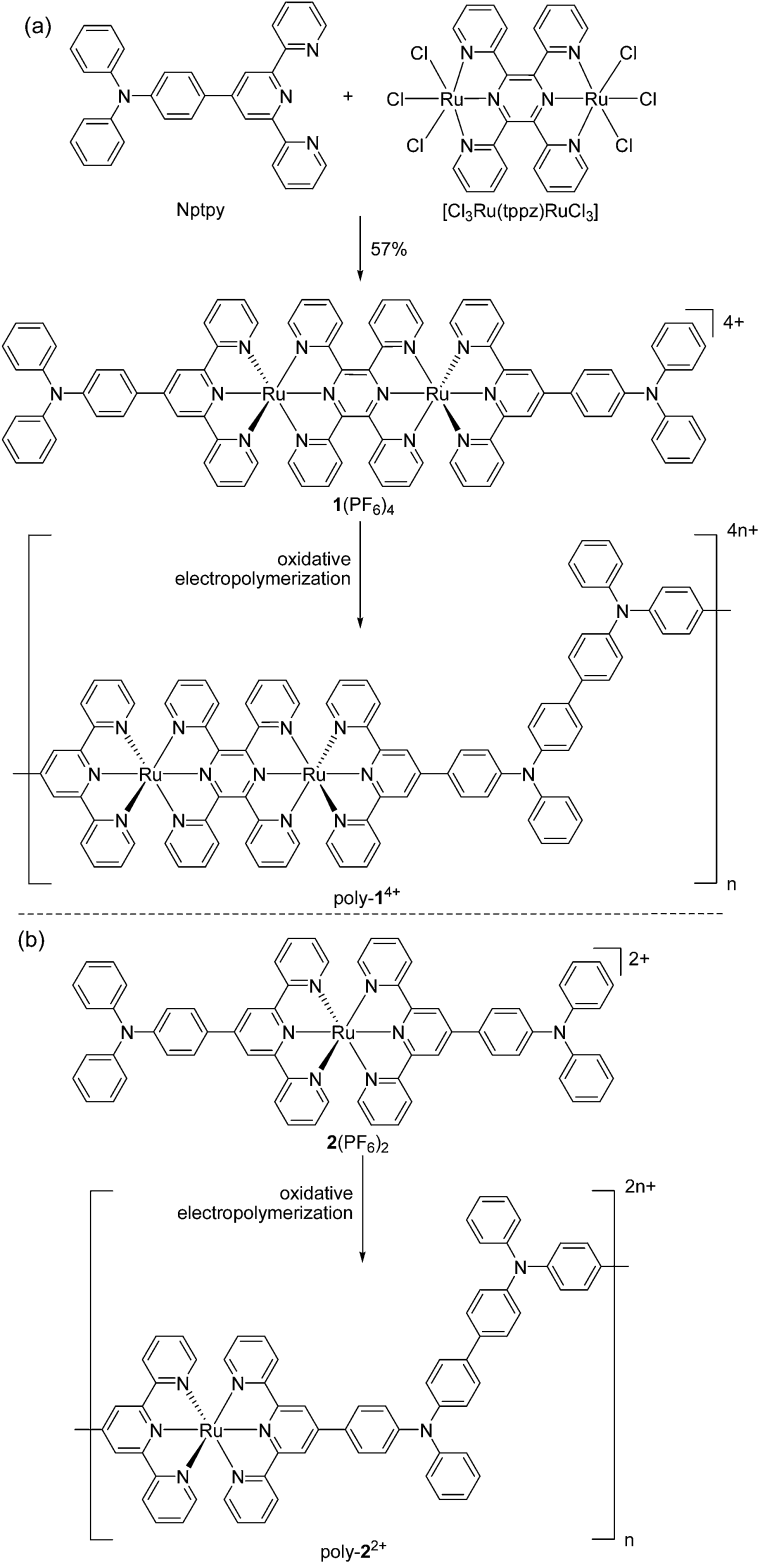
Synthesis of (a) poly-**1** ^4+^ and (b) poly-**2** ^2+^ *via* the oxidative electropolymerization of **1**(PF_6_)_4_ and **2**(PF_6_)_2_, respectively. The counteranions of the polymers are mostly ClO_4_^–^ ions, which were included from the electrolyte during the electropolymerization.

Encouraged by recent work using materials with multiple redox processes for information storage and memory devices,^1b,9,11,12^ we have designed the diruthenium complex **1**(PF_6_)_4_ as a monomer for electropolymerization (Scheme 1a). This complex contains two Ru ions which are bridged by 2,3,5,6-tetrakis(2-pyridyl)pyrazine (tppz) and capped by two triphenylamine-substituted terpyridine ligands. Based on previous studies on the efficient polymerization of triphenylamine-appended ruthenium complexes,^13^ we conjectured that the head-to-tail anodic electropolymerization of **1**(PF_6_)_4_ would afford poly-**1** ^4+^ with alternating tppz-bridged diruthenium and biphenyl-bridged diamine (tetraphenylbenzidine) structural segments. The Ru-chelated tppz ligand is believed to accept multiple electrons easily and significantly lower the energy level of the lowest unoccupied molecular orbital (LUMO) with respect to monoruthenium complexes. As a reference, the prototype diruthenium complex [(tpy)Ru(tppz)Ru(tpy)]^4+^ (tpy = 2,2′:6′,2′′-terpyridine) shows tppz^0/–^ and tppz^2–/–^ processes at –0.39 and –0.86 V *vs.* Ag/AgCl, respectively.^14^ On the other hand, the biphenyl-bridged diamine segment is easily oxidized and the energy level of the highest occupied molecular orbital (HOMO) can be determined by the corresponding N˙^+/0^ potentials. Note that the N˙^+/0^ potential of triphenylamine (0.92 V *vs.* SCE) is significantly decreased after dimerization (0.69 V).^15^

**Fig. 2:**
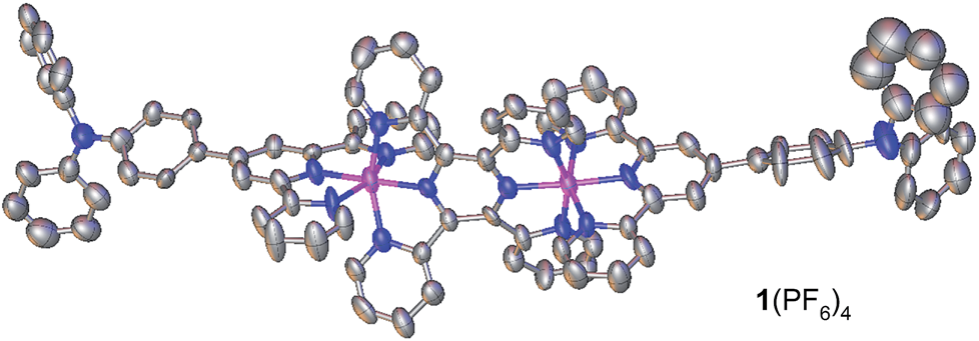
ORTEP drawing of the single-crystal X-ray structure of **1**(PF_6_)_4_ at 30% probability. Anions and H atoms are omitted for clarity. Color code: carbon, grey; nitrogen, blue; pink, ruthenium.

Complex **1**(PF_6_)_4_ was synthesized by the reaction of 4′-(*p-N*,*N*-diphenylamino)phenyl-2,2′:6′,2′′-terpyridine (Nptpy)^13*b*^ with [Cl_3_Ru(tppz)RuCl_3_], followed by anion exchange using KPF_6_ (Scheme 1a). For the purpose of comparison, the monoruthenium complex **2**(PF_6_)_2_ with two appended triphenylamine units was also prepared by the reaction of Nptpy with RuCl_3_, and was later polymerized to give poly-**2** ^2+^ films for device testing (Scheme 1b). The structure of **1**(PF_6_)_4_ was confirmed by single-crystal X-ray analysis (Fig. 2).

**Fig. 3:**
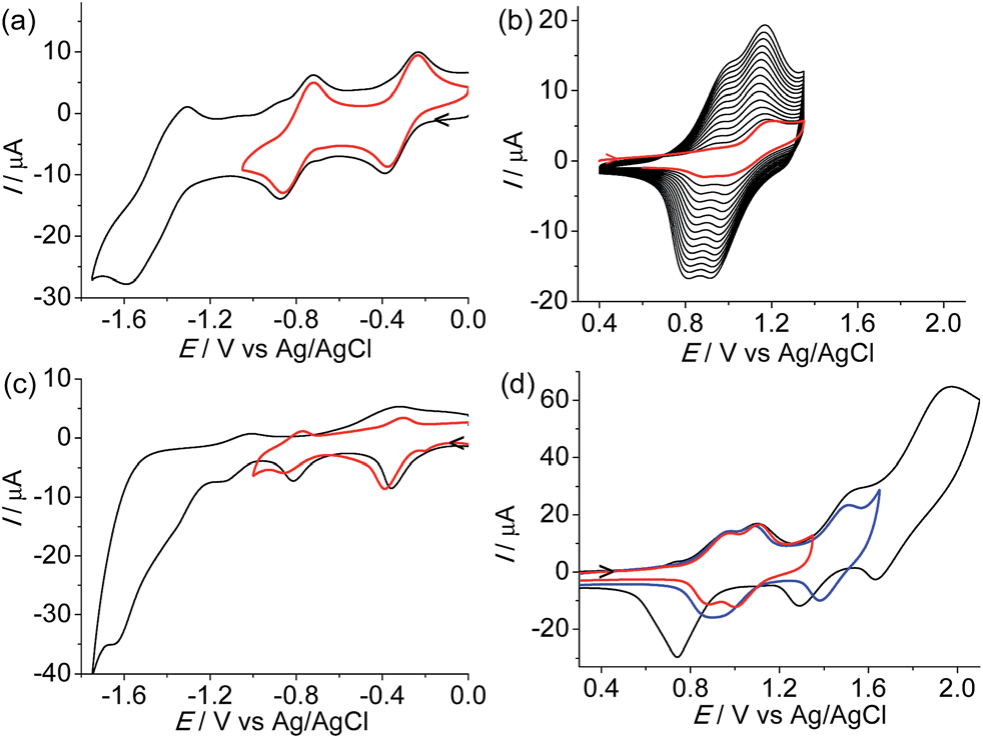
(a) Cathodic CVs of **1**(PF_6_)_4_ at a Pt disk electrode (*d* = 2 mm) in 0.1 M Bu_4_NClO_4_/CH_2_Cl_2_. (b) CVs recorded during repeated potential scans between +0.40 and +1.35 V. (c and d) CVs of the obtained poly-**1** ^4+^/Pt film in a clean electrolyte solution. The scan rates are 100 mV s^–1^.

Complex **1**(PF_6_)_4_ shows two cathodic redox waves at –0.31 and –0.79 V *vs.* Ag/AgCl (Fig. 3a), corresponding to the tppz^0/–^ and tppz^2–/–^ processes, respectively.^14^ The redox peaks at around –1.50 V are due to the reduction of the tpy ligands. In the initial anodic scan, an oxidation peak at +1.18 V was observed (Fig. 3b). When the potential was scanned repeatedly between +0.40 and +1.35 V at a Pt disk electrode, the current in the cyclic voltammogram (CV) increased gradually and continuously with the appearance of two new redox waves. This indicated that the oxidative electropolymerization of **1**(PF_6_)_4_ in CH_2_Cl_2_ proceeded smoothly on the Pt electrode surface.

Fig. 3c and d show the CVs of the obtained poly-**1** ^4+^/Pt film. The cathodic waves are less well-defined with respect to those of the monomer. However, the tppz^0/–^ and tppz^2–/–^ processes of the polymeric materials can be clearly recognized and occur at similar potentials (–0.34 and –0.81 V). In the region between –1.0 and –1.8 V, poly-**1** ^4+^ displays some irreversible or quasi-reversible waves. These waves are due to the reduction of tpy ligands and possibly complicated by some charge-trapping peaks. The anodic scan of the polymer shows four well-defined consecutive waves at +0.90, +1.05, +1.44, and +1.74 V, respectively. The former two waves are due to the N˙^+/0^ processes of the tetraphenylbenzidine segments of the polymers.^13,15^ The latter two peaks are due to the stepwise Ru^III/II^ processes of the diruthenium segment.^14^ Both anodic and cathodic currents are linearly dependent on the scan rate (Fig. S1 in the ESI†), which is characteristic of redox processes confined to electrode surfaces. The first three anodic waves are chemically reversible. When the potential was scanned beyond +2.0 V, a fourth wave at +1.74 V appeared, which was much higher in current with respect to the first three waves. It is possible that further irreversible oxidation of the aminium radical cations is involved in the fourth wave, which causes the return reduction waves to differ significantly from those scanned at voltages no more positive than +1.6 V. On the basis of the electrochemical results, the LUMO and HOMO energy levels of poly-**1** ^4+^ are estimated to be –4.4 and –5.6 eV *vs.* vacuum, respectively.

The presence of the well-defined N˙^+/0^ and Ru^III/II^ redox waves indicates the polymer has the expected linear structure with alternating tetraphenylbenzidine and diruthenium structural segments. The possibility of further chain propagation on the phenyl groups of the tetraphenylbenzidine unit to form a cross-linked structure should be low. Otherwise, the N˙^+/0^ waves would be very complex due to the presence of a strongly-coupled multi-triarylamine structural component. The head-to-tail oxidative electropolymerization mechanism and similar alternating polymer structures have been proposed for other related compounds.^13,16^ The FTIR spectrum of **1**(PF_6_)_4_ shows an intense peak at 843 cm^–1^ due to PF_6_^–^ stretching (Fig. S2†). The poly-**1** ^4+^ sample, obtained by scratching the polymeric film off the electrode surface, shows the disappearance of this signal. Instead, a strong signal at 1089 cm^–1^, assigned to ClO_4_^–^ anions, is observed.^17^ This indicates that the counteranions of poly-**1** ^4+^ are largely ClO_4_^–^ ions, incorporated from the electrolyte (^*n*^Bu_4_NClO_4_) during electropolymerization.

**Fig. 4:**
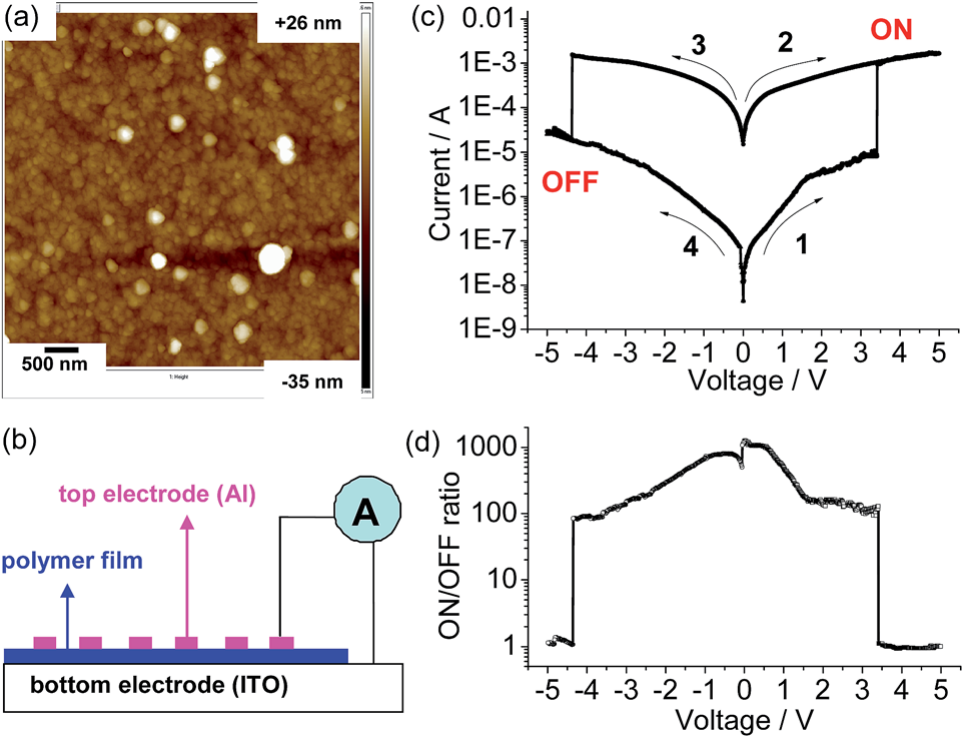
(a) AFM height image of the poly-**1** ^4+^/ITO film (size: 5 μm × 5 μm). (b) Schematic representation of the memory device structure. (c) Typical *I*–*V* characteristics of the ITO/poly-**1** ^4+^/Al device with an active area of 6.0 mm^2^. The arrows denote switching order and direction. The thicknesses of the polymer film and Al electrode are 100 and 80 nm, respectively. (d) Plot of the ON/OFF current ratio *versus* voltage. The *y* axes of (c) and (d) are in logarithmic scale.

A similar electropolymerization process was performed using **1**(PF_6_)_4_ on an indium-tin-oxide (ITO) glass electrode to afford a polymeric thin film for memory device fabrication. Fig. 4a shows the surface morphology of the obtained thin film measured by atomic force microscopy (AFM), which shows a homogenous surface texture with a mean roughness, rms, of 5.5 nm. The thickness of the film was estimated by measuring the step height produced by scanning across a scratching edge (Fig. S3†). X-ray photoelectron spectroscopy (XPS) of the film shows bands for O 1s (532.1 eV), N 1s (400.1 eV), C 1s (284.88 eV), Ru 3d_5_ (281.28 eV), Cl 2p (207.46 eV), and Si 2p (102.05 eV) (Fig. S4†), confirming the presence of the ruthenium and perchlorate ions.

The memory device was fabricated by sandwiching the polymeric film between the substrate ITO electrode and a top layer consisting of an Al electrode (Fig. 4b). The current passing through the polymeric film with an active area of 6.0 mm^2^ was monitored under ambient conditions. Hysteretic current–voltage (*I*–*V*) characteristics were observed for the as-prepared device (Fig. 4c), demonstrating a flash memory function. In the first voltage sweep from 0 to +5 V, an abrupt increase in the current was observed at a switching threshold voltage of +3.4 V. This indicates that the device was switched from a low-conductivity state (OFF state) to a high-conductivity state (ON state), corresponding to the “write” process. The high-conductivity state was retained during the subsequent positive sweep (the second sweep from 0 to +5 V), implying that the data was memorized. One important feature of the present memory device is that the OFF state can be recovered by simply applying a reverse voltage (the third sweep), where an abrupt drop in current occurs at a switching threshold voltage of –4.3 V. This serves as the “erase” process for the memory device. The device remained in the stable OFF state during the fourth sweep from 0 to –5 V right after the erase process. These *I*–*V* characteristics define the electrical bistability of the device. The distinct electrical bistates between –4.3 and +3.4 V allow any voltage in this range to read as an OFF or ON signal depending upon the history of the voltage sweep, with an ON/OFF current ratio ranging from 100–1000 (Fig. 4d). Among fifty devices measured, eight devices displayed such well-defined *I*–*V* characteristics with an ON/OFF current ratio over 100. Circuit shortage is one reason for the low success rate. We hope that the device performance will be further improved by the optimization of device fabrication in the future.

**Fig. 5:**
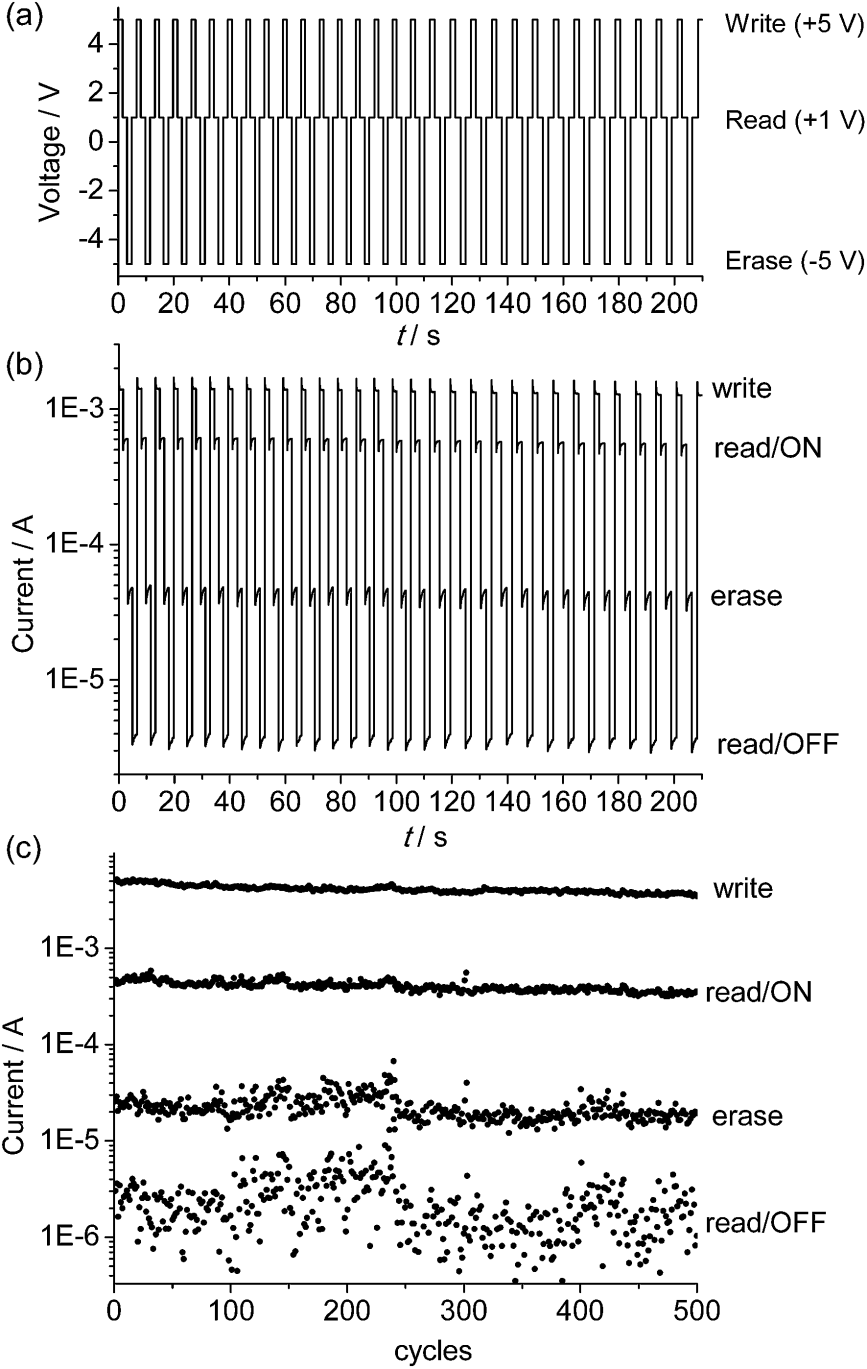
(a) Input applied voltage sequence and (b and c) output current responses during repeated write/read/erase/read (+5 V/1 V/–5 V/1 V) cycles for the ITO/poly-**1** ^4+^/Al device.

The endurance of the above device as a random-access memory (RAM) device was examined by applying repeated write/read/erase/read cycles (+5 V/1 V/–5 V/1 V) in pulse mode (Fig. 5a and b). After a write process at +5 V for 1.5 s, the device was immediately switched to the high-conducting ON state (current = 1.4 × 10^–3^ A), followed by a read process at +1 V for 1.7 s (current = 6.0 × 10^–4^ A). After that, an erase process at –5 V was applied for 1.5 s. The device was then switched to the low-conducting OFF state (current = 4.6 × 10^–5^ A), which was again read out at +1 V for 1.7 s (current = 3.8 × 10^–6^ A). No resistance degradation was observed when the device was tested for 500 write/read/erase/read cycles under ambient conditions, with an ON/OFF ratio over 200 (Fig. 5c). This suggests that the device using the poly-**1** ^4+^ film has good stability and reproducibility.

**Fig. 6:**
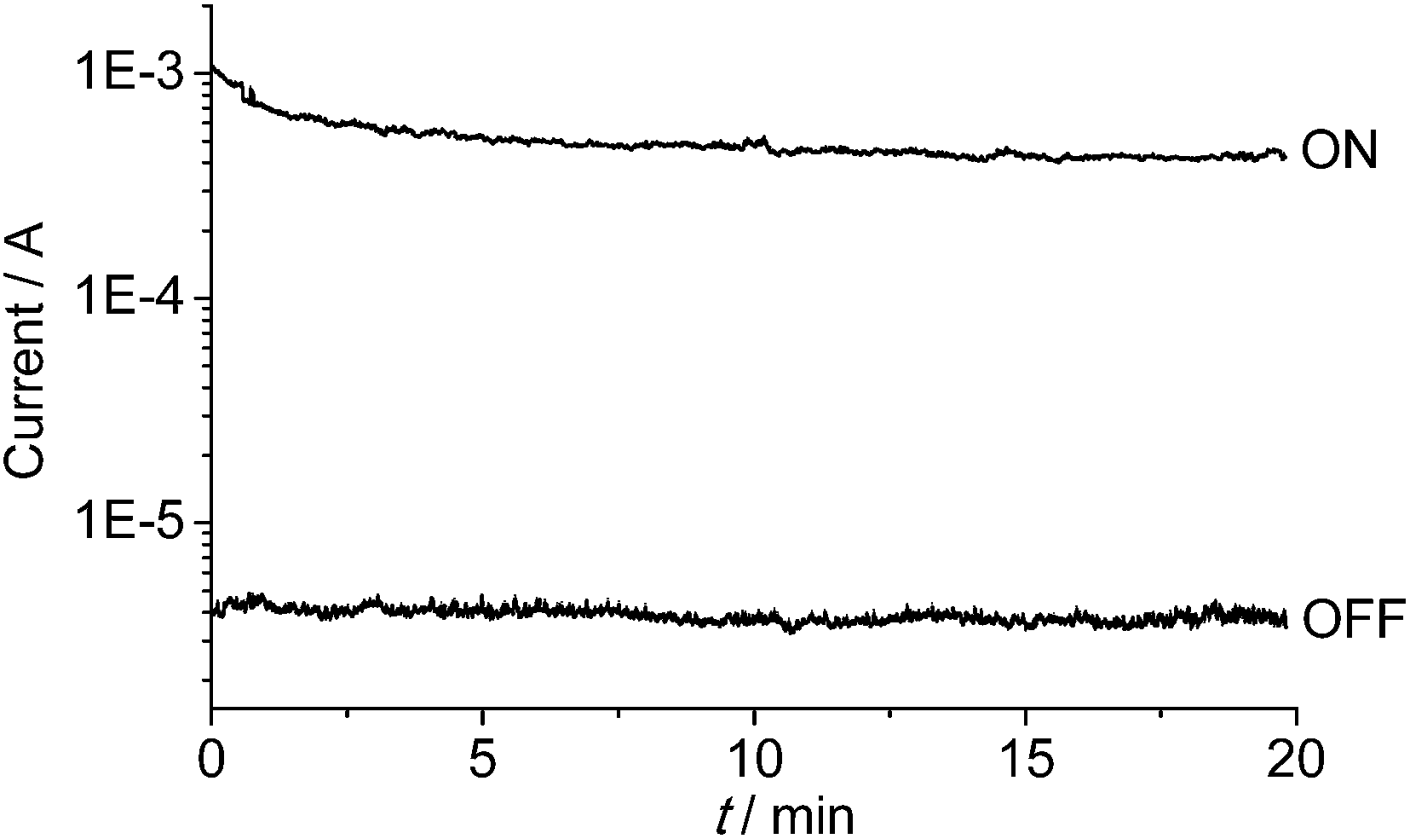
Retention times of the ON- and OFF-state data under a readout voltage of +1 V. The ON and OFF states were induced by +5 and –5 V, respectively.

The memory device displays a long retention time (Fig. 6). When the device was turned ON or OFF by applying a voltage greater than the threshold value, the high- or low-conducting state was retained after 20 min under a small readout voltage (+1 V). During the test of the retention time for the ON state, the current dropped a little in the first 3 min, and then remained at a steady state. It is possible that current is consumed during the first few minutes to reach a more balanced conducting state.

**Fig. 7:**
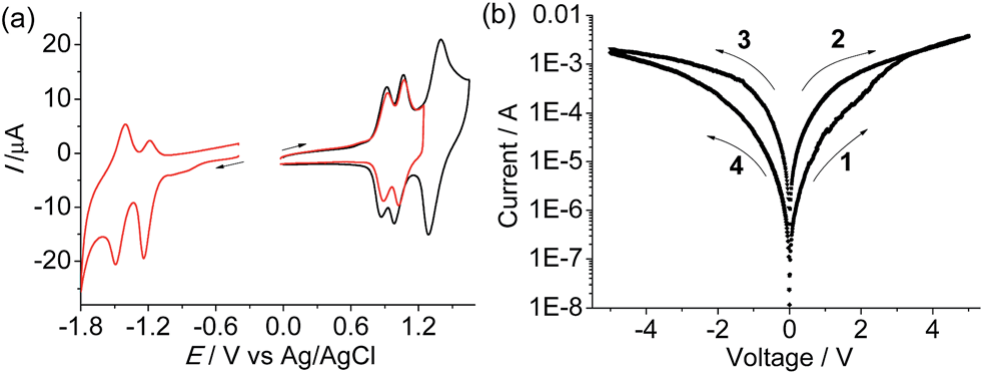
(a) CVs of the poly-**2** ^2+^/Pt film in a clean electrolyte solution at 100 mV s^–1^. (b) Typical *I*–*V* characteristics of the ITO/poly-**2** ^2+^/Al device with a logarithmic scale for the current. The arrows denote switching order and direction.

The monoruthenium complex **2**(PF_6_)_2_ was polymerized by a similar electrochemical oxidation method (Fig. S5†). Poly-**2** ^2+^ displays similar N˙^+/0^ processes to poly-**1** ^4+^ at +0.91 and +1.04 V *vs.* Ag/AgCl (the peak at +1.44 V is due to the Ru^III/II^ process, Fig. 7a), which means that these two polymers have similar HOMO levels. Two cathodic redox waves at –1.21 and –1.45 V are observed for poly-**2** ^2+^, associated with two tpy^0/–^ processes. The LUMO of poly-**2** ^2+^ is estimated to be –3.5 eV *vs.* vacuum, which is 0.9 eV more destabilized with respect to that of poly-**1** ^4+^. A typical AFM height image of the poly-**2** ^2+^/ITO film is given in Fig. S6.† The sandwiched ITO/poly-**2** ^2+^/Al device shows much poorer memory performance with respect to the poly-**1** ^4+^ device. Hysteretic *I*–*V* characteristics were observed for the device with the poly-**2** ^2+^ film (Fig. 7b). However, no abrupt decrease or increase in current occurred, and the best ON/OFF ratio achieved was less than 15. This indicates that the presence of the bridged diruthenium structure in poly-**1** ^4+^ is crucial for the excellent memory function.

**Fig. 8:**
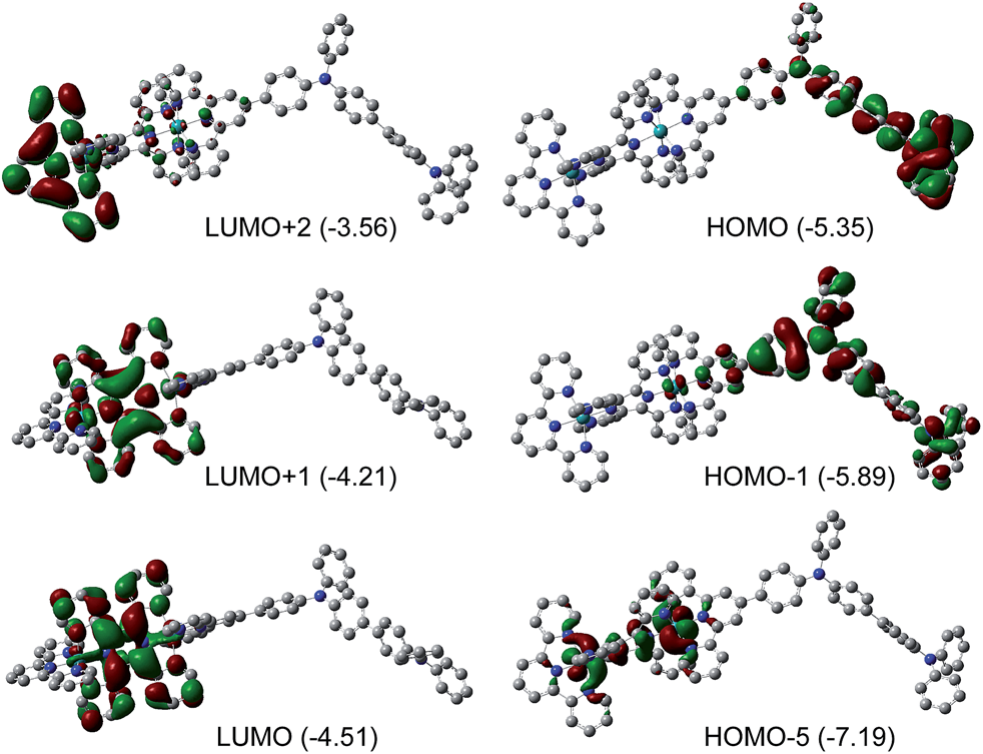
Isodensity plots of the frontier molecular orbitals of the diruthenium-tetraphenylbenzidine basic structural component of poly-**1** ^4+^. DFT methods: B3LYP/LANL2DZ/6-31-G*/CPCM. Eigenvalues in eV are shown in parentheses for each energy level.

We propose that the mechanism of the field-induced conductivity of the poly-**1** ^4+^ film probably involves the formation of a charge transfer state.^18^ DFT calculations for the basic diruthenium-tetraphenylbenzidine structure of poly-**1** ^4+^ show that the HOMO and LUMO energy levels are localized on the tetraphenylbenzidine unit and the tppz bridging ligand, respectively (Fig. 8). The Ru ions and tpy ligands play more important roles in lower occupied orbitals (*e.g.*, HOMO–5) and higher unoccupied orbitals (*e.g.*, LUMO+2), respectively. A high electric field may facilitate intermolecular or intramolecular charge transfer from the tetraphenylbenzidine donor to the tppz acceptor, resulting in a high-conducting state with a high concentration of charge carriers. For poly-**2** ^2+^ with a much higher LUMO energy level, the formation of such a charge transfer state is difficult, and the corresponding device displays much poorer memory performance. DFT calculations for the basic monoruthenium-tetraphenylbenzidine structure of poly-**2** ^2+^ show that the energy gap between the tetraphenylbenzidine-dominated HOMO and the tpy-localized LUMO is 2.15 eV (Fig. S7†), which is much larger relative to that of the basic diruthenium-tetraphenylbenzidine structure of poly-**1** ^4+^ (0.84 eV). This also suggests that the formation of a charge transfer state for poly-**2** ^2+^ is much more difficult with respect to poly-**1** ^4+^.

## Conclusions

In summary, we have demonstrated an electropolymerized film-based single-layer electrical device that displays excellent resistive memory performance, including a high ON/OFF ratio, low operational voltage, good stability, and long retention time. We believe that the performance of the device can be further improved by capsulation and device optimization. Compared to spin-coating and vacuum deposition, film formation by electropolymerization is a much more convenient and cheaper method. Considering that a large number of redox-active electropolymerized films of both organic and organometallic monomers are available to date,^10^ our work demonstrates an important alternative for the development of high-density memristor materials and devices with respect to those based on spin-coating or vacuum deposition of small molecules or organic polymers. In addition, this work represents another successful yet rare example of using transition metal complexes as the active layer for memristor devices.^7–9^ The polymeric films with the diruthenium complex exhibit much better memory performance with respect to those with the monoruthenium complex. The high HOMO energy level and low LUMO energy level arising respectively from the tetraphenylbenzidine and ruthenium-chelated tppz units in poly-**1** ^4+^ are crucial to the successful memory performance. This highlights the critical roles of the molecular design and electronic properties of materials in developing excellent molecular devices.

## Experimental section

### Electrochemical measurements

All electrochemical measurements were taken using a CHI 660D potentiostat under an atmosphere of nitrogen. All measurements were carried out in 0.1 M ^*n*^Bu_4_NClO_4_ in denoted solvents. The potentials are referenced to a Ag/AgCl electrode in saturated aqueous NaCl, ignoring the liquid junction potential. The working electrode was a home-made Pt disk electrode (*d* = 2 mm) or a transparent ITO glass electrode (<10 Ω per square). The ITO glass was pre-cleaned with water, acetone, and then 2-propanol in an ultrasonic bath (15 min each), and dried in a nitrogen airflow before use. A large area platinum wire coil was used as the counter electrode. A three-compartment electrochemical cell was used in the electropolymerization experiments. The working electrode (ITO glass) was positioned parallel to and opposite the counter electrode.

### X-ray crystallography

The X-ray diffraction data were collected using a Rigaku Saturn 724 diffractometer on a rotating anode (Mo–K radiation, 0.71073 Å) at 173 K. The structure was solved by the direct method using SHELXS-97 (ref. 19) and refined with Olex2.^20^ A single crystal of **1**(PF_6_)_4_ was obtained by slow diffusion of hexane into a solution in dichloromethane. Crystallographic data for **1**(PF_6_)_4_ (CCDC ; 1023945): C_90_H_64_N_14_Ru_2_F_24_P_4_, *M* = 2123.57, triclinic, space group *P*1, *a* = 13.811(3), *b* = 18.252(4), *c* = 20.803(3) Å, *α* = 84.81°, *β* = 82.76°, *γ* = 72.33°, *U* = 3669.3(13) Å^3^, *T* = 173 K, *Z* = 2, radiation type MoKα, radiation wavelength 0.71073 Å, final *R* indices *R*_1_ = 0.1354, w*R*_2_ = 0.3591, *R* indices (all data) *R*_1_ = 0.1588, w*R*_2_ = 0.3829.

### XPS measurements

XPS spectroscopy data were obtained with an ESCALab220i-XL electron spectrometer from VG Scientific using 300 W Al Kα radiation. The base pressure was about 3 × 10^–9^ mbar. The binding energies were referenced to the C 1s line at 284.8 eV from adventitious carbon.

### AFM images

AFM was carried out with a Brucker Multimode 8 using tapping-mode with a scan speed of 1 Hz.

### Fabrication and characterization of memory devices

An 80 nm-thick aluminum top electrode was thermally evaporated onto the polymer film on ITO glass at a pressure of around 10^–6^ Torr. The active area of the film sandwiched between two electrodes was 2.0 mm × 3.0 mm in size. The devices were characterized under ambient conditions, using a Keithley 4200 SCS semiconductor parameter analyzer.

### Computational methods

DFT calculations were carried out using the B3LYP exchange correlation functional^21^ and implemented in the Gaussian 09 package.^22^ The electronic structures of the complexes were determined using a general basis set with the Los Alamos effective core potential LANL2DZ basis set for ruthenium and 6-31G* for other atoms.^23^ Solvation effects in CH_2_Cl_2_ were included by using the conductor-like polarizable continuum model (CPCM).^24^ No symmetry constraints were used in the optimization (nosymm keyword was used). Frequency calculations have been performed with the same level of theory to ensure that the optimized geometries were local minima. All orbitals have been computed at an isovalue of 0.02 e per bohr^3^.

### Synthesis

NMR spectra were recorded in the designated solvent on a Bruker Avance 400 MHz spectrometer. Spectral shifts are reported in ppm values from the residual protons of the deuterated solvent. Mass data were obtained using a Bruker Daltonics Inc. Apex II FT-ICR or Autoflex III MALDI-TOF mass spectrometer. The matrix for MALDI-TOF measurement was α-cyano-4-hydroxycinnamic acid. Microanalysis was carried out using a Flash EA 1112 or Carlo Erba 1106 analyzer at the Institute of Chemistry, Chinese Academy of Sciences.

#### Synthesis of complex **1**(PF_6_)_4_

Ligand Nptpy (0.12 mmol, 57 mg), [Cl_3_Ru(tppz)RuCl_3_]^25^ (0.05 mmol, 40 mg), ethanol (10 mL), and NEt_3_ (5 mL) were added to a reaction flask. The mixture was bubbled with nitrogen for 10 min, followed by refluxing for 10 h. After cooling to room temperature, the solvent was removed under reduced pressure. The residue was dissolved in 2 mL of methanol, followed by the addition of an excess of aq. KPF_6_. The resulting precipitate was collected by filtering and washing with water and Et_2_O. The obtained solid was purified by chromatography on silica gel (eluent: CH_3_CN/H_2_O/aq. KNO_3_, 100/10/0.1), followed by anion exchange using KPF_6_, to give 61 mg of **1**(PF_6_)_4_ as a purple solid in 57% yield. ^1^H NMR (400 MHz, CD_3_CN): *δ* 7.27–7.36 (m, 20H), 7.45–7.52 (m, 12H), 7.75 (d, *J* = 5.2 Hz, 4H), 7.86 (d, *J* = 5.2 Hz, 4H), 7.97 (t, *J* = 8.0 Hz, 4H), 8.10 (t, *J* = 7.6 Hz, 4H), 8.21 (d, *J* = 4.4 Hz, 4H), 8.78 (d, *J* = 8.0 Hz, 4H), 8.99 (d, *J* = 8.4 Hz, 4H), 9.13 (s, 4H). ^13^C NMR (100 MHz, CD_3_CN): *δ* 121.7, 122.0, 125.4, 125.5, 126.4, 128.2, 128.7, 129.4, 129.9, 130.1, 130.5, 138.4, 139.6, 147.4, 150.0, 150.4, 151.3, 154.0, 154.7, 155.2, 155.6, 158.4. MALDI-MS: 1688.1 for [M – 3PF_6_]^+^, 1544.1 for [M – 4PF_6_]^+^, 966.0 for [M – 4PF_6_ – Ru(Nptpy)]^+^, anal. calcd for C_90_H_64_F_24_N_14_P_4_Ru_2_·3H_2_O: C, 49.64; H, 3.24; N, 9.01; found: C, 49.45; H, 3.16; N, 8.86.

#### Synthesis of complex **2**(PF_6_)_2_

This complex was prepared according to a slightly modified known procedure.^26^ Ligand Nptpy (0.10 mmol, 48 mg), RuCl_3_·3H_2_O (0.050 mmol, 13 mg), EtOH (10 mL), H_2_O (2 mL), and NEt_3_ (5 mL) were added to a round-bottom flask. The mixture was refluxed under a nitrogen atmosphere for 10 h. After cooling to room temperature, the solvent was removed under reduced pressure. The residue was dissolved in 2 mL ethanol, followed by the addition of an excess of aq. KPF_6_. The resulting precipitate was collected by filtering and washing with water and Et_2_O. The crude solid was purified by chromatography on silica gel (eluent: CH_3_CN/H_2_O/aq. KNO_3_, 100/10/0.1), followed by anion exchange using KPF_6_, to give 44 mg of **2**(PF_6_)_2_ as a red solid in 65% yield. ^1^H NMR (400 MHz, CD_3_CN): *δ* 7.16 (t, *J* = 6.8 Hz, 4H), 7.20–7.28 (overlapped, 16H), 7.41–7.45 (overlapped, 12H), 7.92 (t, *J* = 8.0 Hz, 4H), 8.09 (d, *J* = 8.4 Hz, 4H), 8.61 (d, *J* = 8.8 Hz, 4H), 8.94 (s, 4H). MALDI-MS: 1199.3 for [M – PF_6_]^+^, 1054.3 for [M – 2PF_6_]^+^.
