# Supplementary material for: Tuning of resistive memory switching in electropolymerized metallopolymeric films
Source: Chem Sci. 2014 Nov 24;6(2):1308–15. doi: 10.1039/c4sc03345k (PMC5811141; doi:10.1039/c4sc03345k)

Electronic Supplementary Material (ESI) for Chemical Science.
This journal is © The Royal Society of Chemistry 2014

Supporting Information for:

Tuning of Resistive Memory Switching in Electropolymerized Metallopolymeric films

Bin-Bin Cui,[†] Zupan Mao,[‡] Yuxia Chen,[†] Yu-Wu Zhong,[,†] Gui Yu,*[,‡] Chuanlang Zhan,*[,†] and Jiannian Yao*[,†]*

[†]Beijing National Laboratory for Molecular Sciences, CAS Key Laboratory of Photochemistry, Institute of Chemistry, Chinese Academy of Sciences, Beijing 100190, China

[‡]Key Laboratory of Organic Solids, Institute of Chemistry, Chinese Academy of Sciences, Beijing 100190, China

E-mail: zhongyuwu@iccas.ac.cn; yugui@iccas.ac.cn; clzhan@iccas.ac.cn; jnyao@iccas.ac.cn

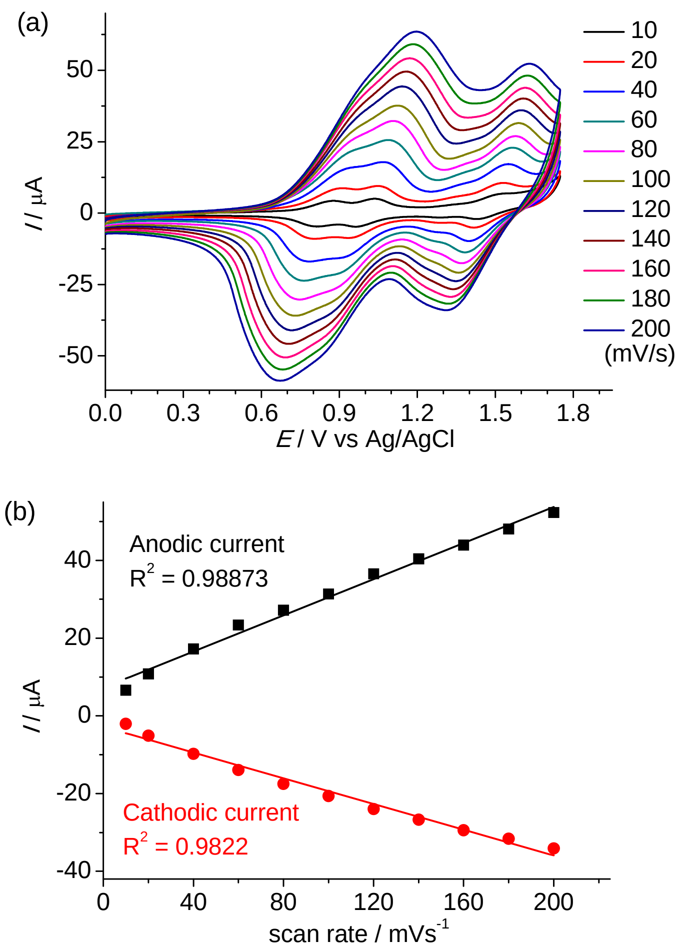

Figure S1. (a) CVs of the poly-**1**$^{4+}$/Pt film at different scan rate. (b) The linear relationship of the currents of the redox wave at +1.44 V versus the scan rate.

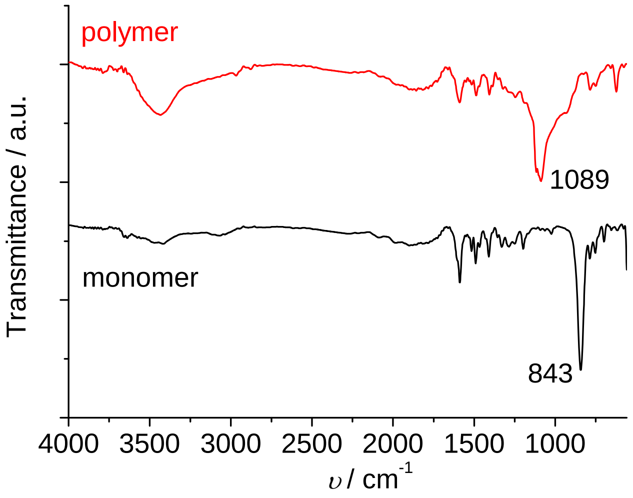

Figure S2. FTIR spectra of **1**$(PF_6)_4$ (black curve) and poly-$\mathbf{1}^{4+}$ (red curve).

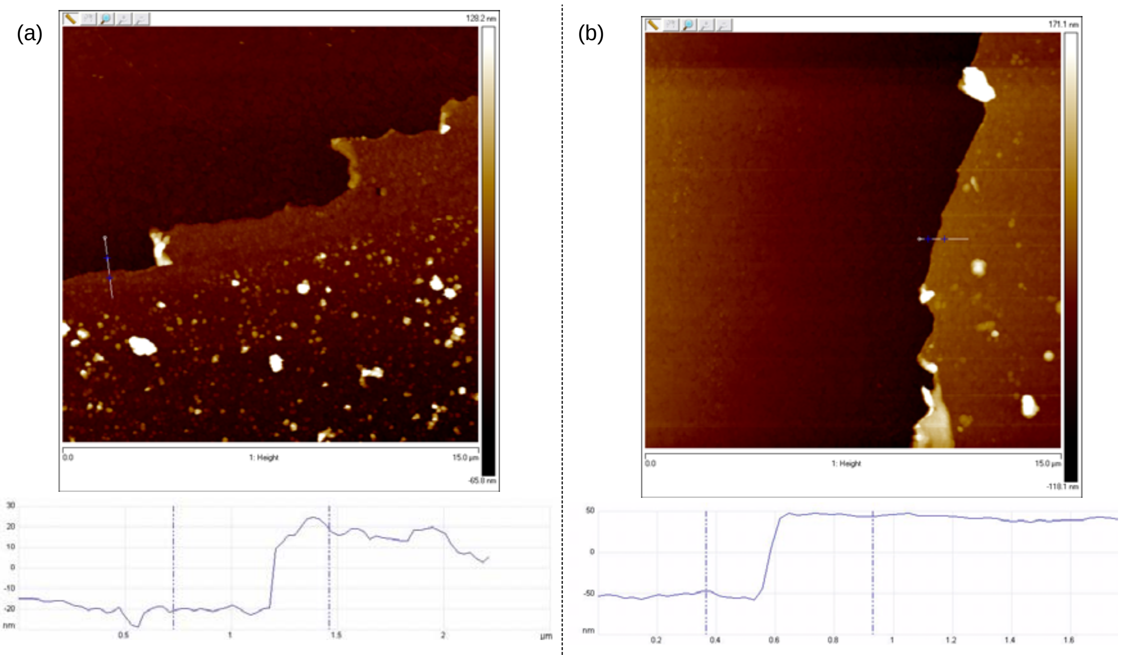

Figure S3. Estimating the thickness of the poly-$\mathbf{1}^{4+}$/ITO film by measuring the step height produced by scanning across a scratching edge using AFM. (a) 40 nm. (b) 100 nm.

(a):

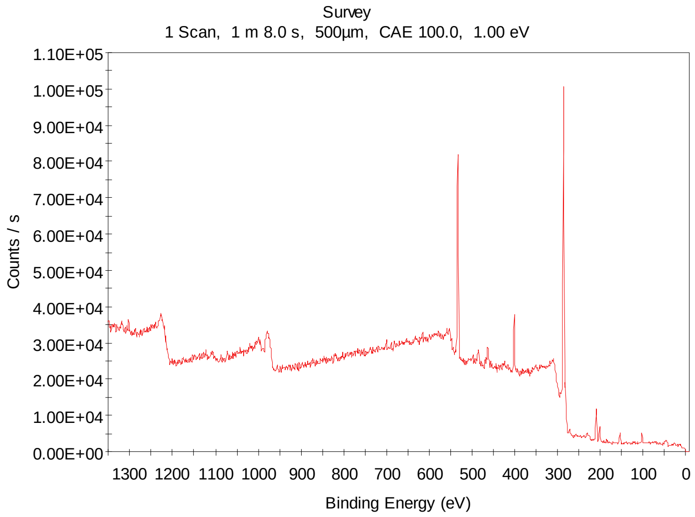

(b):

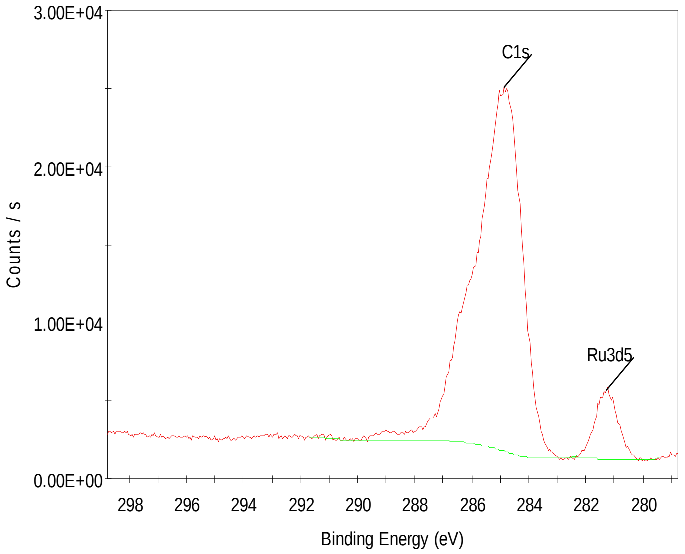

(c):

	Binding energy (eV)	Atomic%
O 1s	532.1	16.1
N 1s	400.1	7.25
C 1s	284.88	69.77
Ru $3d_5$	281.28	0.73
Cl 2p	207.46	4.03
Si 2p	102.05	2.12

Figure S4. XPS survey spectra of poly-**1**$^{4+}$/ITO (black curve) film. (a) Survey scan. (b) Cl and Ru scan. (c) Atomic composition.

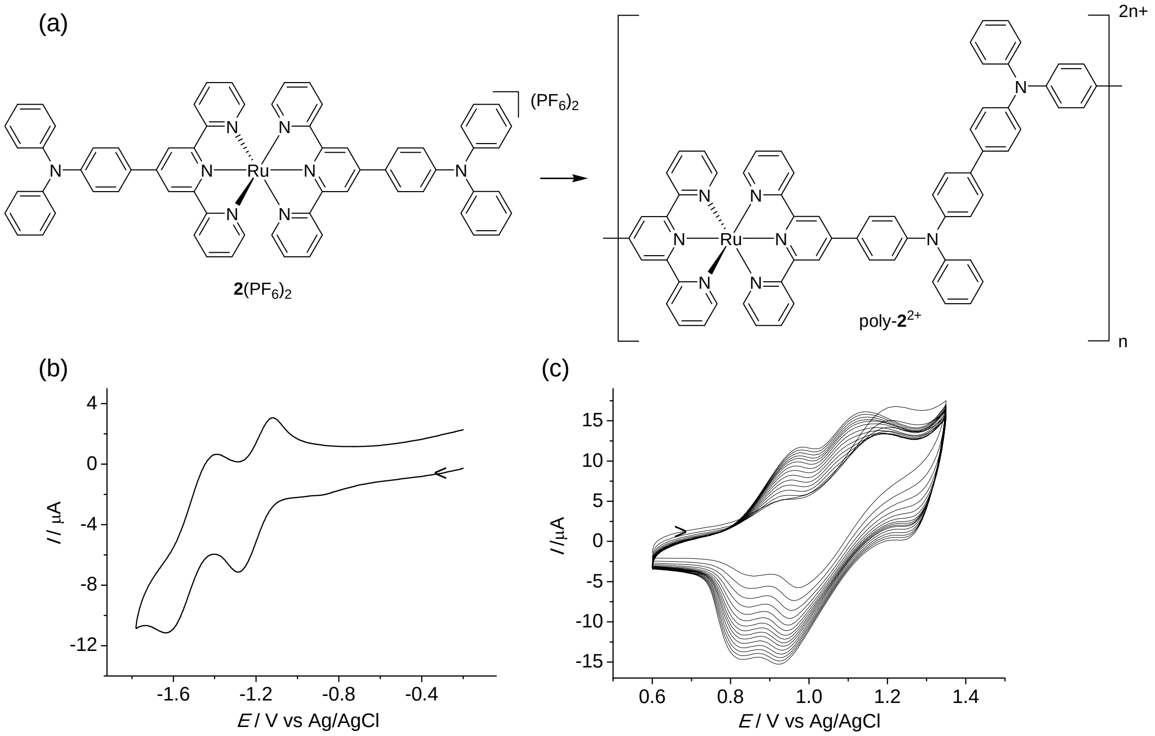

Figure S5. (a) Synthesis of poly-$\mathbf{2}^{2+}$ via the oxidative electropolymerization of **2**(PF_6)$_2$. (b) Cathodic CV of **2**(PF_6)$_2$ at a Pt disk electrode (d = 2 mm) in 0.1 M Bu_4NClO_4/CH_2Cl_2. (c) CVs recorded during repeated potential scan between +0.60 and +1.30 V, which indicates that the electropolymerization of **2**(PF_6)$_2$ occurred smoothly.

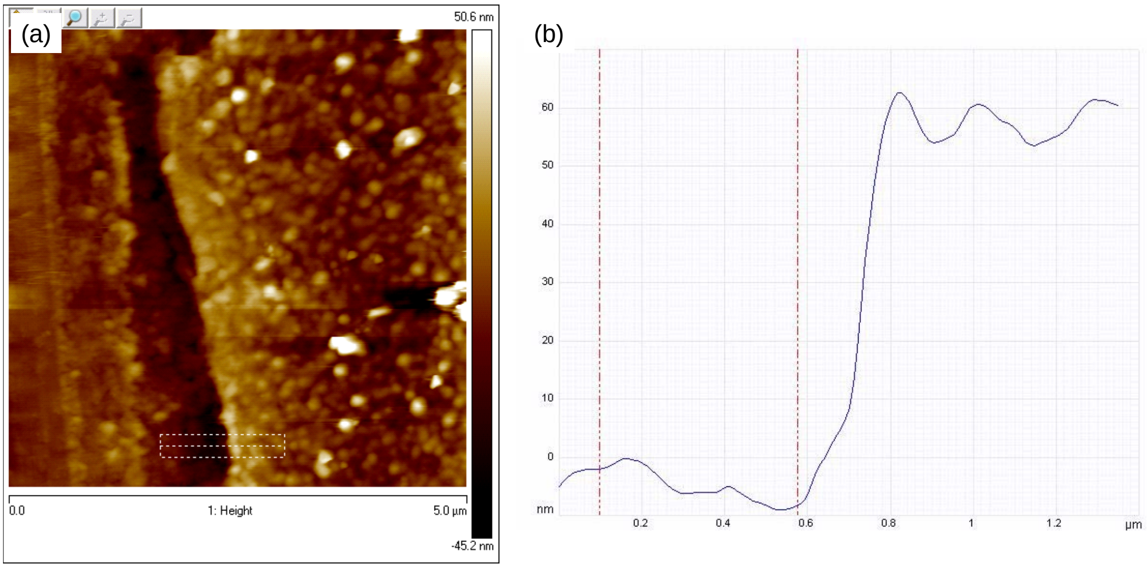

Figure S6. (a) AFM height image of poly-$\mathbf{2}^{2+}$/ITO film and (b) estimating the thickness by a scratching method.

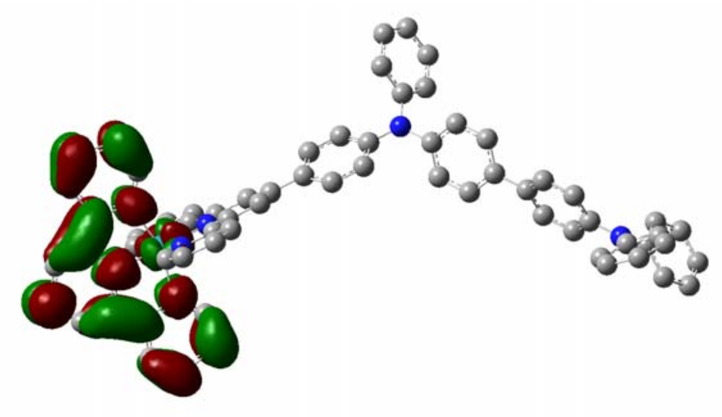

LUMO (-3.03 eV)

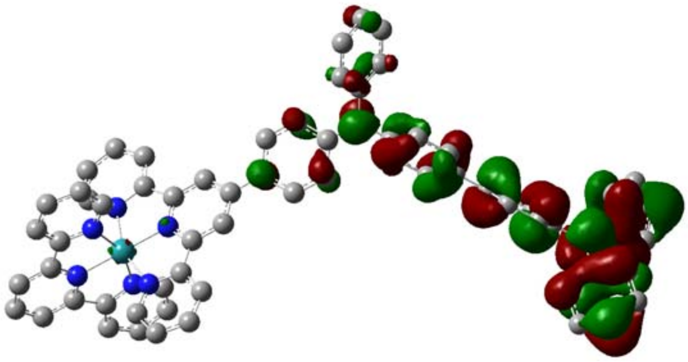

HOMO (-5.18 eV)

Figure S7. Isodensity plots of the frontier molecular orbitals of a monoruthenium-tetraphenylbenzidine basic structural component of poly-$\mathbf{2}^{2+}$. DFT methods: B3LYP/LANL2DZ/6-31-G*/CPCM. Eigenvalues in eV are shown in the parenthesis for each energy level. The HOMO-LUMO gap is 2.15 eV.

Full list of ref 22:

Frisch, M. J.; Trucks, G. W.; Schlegel, H. B.; Scuseria, G. E.; Robb, M. A.; Cheeseman, J. R.; Montgomery, J. A.; Vreven, Jr. T.; Kudin, K. N.; Burant, J. C.; Millam, J. M.; Iyengar, S. S.; Tomasi, J.; Barone, V.; Mennucci, B.; Cossi, M.; Scalmani, G.; Rega, N.; Petersson, G. A.; Nakatsuji, H.; Hada, M.; Ehara, M.; Toyota, K.; Fukuda, R.; Hasegawa, J.; Ishida, M.; Nakajima, T.; Honda, Y.; Kitao, O.; Nakai, H.; Klene, M.; Li, X.; Knox, J. E.; Hratchian, H. P.; Cross, J. B.; Adamo, C.; Jaramillo, J.; Gomperts, R.; Stratmann, R. E.; Yazyev, O.; Austin, A. J.; Cammi, R.; Pomelli, C.; Ochterski, J. W.; Ayala, P. Y.; Morokuma, K.; Voth, G. A.; Salvador, P.; Dannenberg, J. J.; Zakrzewski, V. G.; Dapprich, S.; Daniels, A. D.; Strain, M. C.; Farkas, O.; Malick, D. K.; Rabuck, A. D.; Raghavachari, K.; Foresman, J. B.; Ortiz, J. V.; Cui, Q.; Baboul, A. G.; Clifford, S.; Cioslowski, J.; Stefanov, B. B.; Liu, G.; Liashenko, A.; Piskorz, P.; Komaromi, I.; Martin, R. L.; Fox, D. J.; Keith, T.; Al-Laham, M. A.; Peng, C. Y.; Nanayakkara, A.; Challacombe, M.; Gill, P. M. W.; Johnson, B.; Chen, W.; Wong, M. W.; Gonzalez, C.; Pople, J. A. Gaussian 09, revision A.2; Gaussian, Inc.: Wallingford CT, 2009.

Cartesian coordinates of the DFT-optimized diruthenium-bistriphenylamine structural component of poly-**1**$^{4+}$:
Charge = 4; multiplicity=1

Ru	-2.67466596	4.64354626	10.97733900
N	-2.46744029	6.76266495	10.97054204
N	-2.46446647	2.66122849	11.73168816
N	-4.69045373	4.78957688	11.59694005
N	-3.64652940	4.31708228	9.23898618
N	-1.05837650	4.36741190	9.64111861
C	-2.88212064	7.62371618	10.02630663
H	-3.38782332	7.19132762	9.17111433
C	-2.68154320	8.99804358	10.12190027
H	-3.03732775	9.64822271	9.33080569
C	-2.02366795	9.50256408	11.24121520
H	-1.84860483	10.56749551	11.35061423
C	-1.58936643	8.61721726	12.22563178
H	-1.07446019	8.99082867	13.10237132
C	-1.81960603	7.24886903	12.07565559
C	-1.39448591	6.23968140	13.06909549
C	-0.73162620	6.48764232	14.27368553
H	-0.47321234	7.49616272	14.57190178
C	-0.40462813	5.40892265	15.09591378
C	-0.73756514	4.10748359	14.71945508
H	-0.48300721	3.27318235	15.36129326
C	-1.40216771	3.90674485	13.50703539
C	-1.83407953	2.60854368	12.94715575
C	-1.62694841	1.38972005	13.59429936
H	-1.12908991	1.36103964	14.55589597
C	-2.06456105	0.20852444	12.99898453
H	-1.90735196	-0.74289011	13.49579826
C	-2.70274750	0.27220044	11.76252707
H	-3.05931937	-0.61995810	11.26050021
C	-2.88229890	1.51690856	11.16505913
H	-3.37455614	1.60854008	10.20422242
C	-5.13993038	5.20226134	12.79293059
H	-4.39269512	5.32118331	13.56846715
C	-6.48196801	5.48582666	13.03579051
H	-6.79041034	5.81670563	14.02092926
C	-7.39061768	5.36419833	11.98799182
H	-8.43703653	5.61410568	12.12532766
C	-6.93623122	4.92070228	10.74764131
H	-7.62525561	4.84256681	9.91693945
C	-5.58678711	4.60020137	10.57527251
C	-4.99766492	4.18198107	9.28468159
C	-2.90848587	4.23919853	8.10131326
C	-1.45551855	4.11847026	8.35128394
C	-0.53830152	3.65340707	7.40478524
H	-0.87003114	3.38368543	6.41070990
C	0.80421160	3.51340295	7.75050319
H	1.51856981	3.15276882	7.01839567
C	1.20559779	3.83520079	9.04419826
H	2.24034752	3.75178764	9.35629462
C	0.23974385	4.24374684	9.96110151
H	0.50090195	4.46546618	10.98901715
N	-4.06228008	4.28528425	4.51349171
N	-4.93110683	3.86409054	6.94993384
N	-7.33849398	2.88079258	6.63246686

C	-3.75955068	4.62543737	3.25033817
H	-4.47227786	4.32440083	2.49183727
C	-2.61126395	5.34065275	2.91796945
H	-2.41555239	5.58948851	1.88120128
C	-1.75695489	5.74534063	3.94025617
H	-0.87425927	6.33854711	3.72731877
C	-2.05438363	5.38338493	5.25241335
H	-1.41353403	5.71063491	6.06067471
C	-3.19288059	4.61849626	5.52188913
C	-3.62553331	4.23599157	6.88333043
C	-5.62391826	3.71055834	8.10912862
C	-6.91924061	3.01905149	7.93201480
C	-7.62870871	2.41443432	8.97367523
H	-7.25299318	2.45839586	9.98745602
C	-8.81247403	1.73179155	8.70163558
H	-9.36577801	1.26207789	9.50760084
C	-9.26281657	1.65848528	7.38617010
H	-10.18266607	1.14652874	7.12755854
C	-8.48778325	2.23314898	6.38155953
H	-8.77827923	2.16452640	5.33998525
Ru	-5.89881224	3.50886554	5.21813654
N	-6.89145182	5.36412346	4.86776160
N	-5.33301879	1.49495767	4.80351606
C	-6.85864585	6.46590859	5.63634805
H	-6.24343565	6.41037620	6.52662406
C	-7.56692806	7.62256057	5.32381116
H	-7.50506131	8.48362620	5.97949685
C	-8.34185961	7.64001103	4.16618236
H	-8.90909666	8.52244664	3.88981036
C	-8.37949602	6.50355669	3.36136049
H	-8.97554873	6.49805992	2.45689767
C	-7.64767050	5.37223574	3.72500056
C	-7.62432183	4.12496632	2.92847068
C	-8.28923754	3.90324401	1.72735529
H	-8.91816057	4.67854573	1.31005537
C	-8.17047000	2.66308254	1.06329405
C	-7.36515923	1.67834733	1.67539724
H	-7.22081544	0.72332768	1.18768798
C	-6.72845454	1.94053061	2.88352480
C	-5.85960667	1.00815513	3.63589127
C	-5.57700591	-0.29146633	3.21288588
H	-6.00056229	-0.66717347	2.28939564
C	-4.74899721	-1.10503247	3.98307812
H	-4.52493613	-2.11605645	3.65980554
C	-4.21876716	-0.60008663	5.16834481
H	-3.57076643	-1.19640266	5.80054715
C	-4.53649631	0.70268042	5.54085723
H	-4.14591385	1.13368986	6.45520561
C	-8.85211899	2.40912970	-0.21451111
C	-9.18740998	1.10244140	-0.62479679
C	-9.19637678	3.46165864	-1.08730523
C	-9.82327142	0.85633675	-1.83126650
H	-8.96873626	0.25483151	0.01750321
C	-9.83090480	3.22906185	-2.29696328
H	-8.94548014	4.48682909	-0.83269703
C	-10.15973703	1.91616418	-2.70334335
H	-10.06143897	-0.16414666	-2.10711235
H	-10.07568807	4.06788369	-2.93772571

C	-10.68428138	2.59863078	-5.01125315
C	-9.43282482	3.06806755	-5.43528912
C	-11.83340544	3.02689858	-5.68989190
C	-9.34017339	3.96165259	-6.49847680
H	-8.53309242	2.73848397	-4.92473845
C	-11.72977285	3.90469638	-6.76505365
H	-12.80733670	2.66166350	-5.37907234
C	-10.48359774	4.40014156	-7.19262797
H	-8.36076473	4.33033928	-6.78819267
H	-12.63412703	4.19975031	-7.28861347
C	-11.56934425	0.48897046	-4.13481030
C	-12.58974208	0.13617547	-3.23966712
C	-11.32743674	-0.31130212	-5.26002806
C	-13.34754983	-1.01368577	-3.46409236
H	-12.78695416	0.76452995	-2.37647392
C	-12.09963568	-1.45099992	-5.48563165
H	-10.53763023	-0.03624965	-5.95215717
C	-13.10874680	-1.80972901	-4.58781531
H	-14.13674213	-1.27813024	-2.76584732
H	-11.90449369	-2.06417335	-6.36090379
H	-13.70589337	-2.69966173	-4.76375632
N	-10.79357517	1.67485074	-3.92450723
C	-10.37950471	5.34638670	-8.32919158
C	-11.38458704	6.29685937	-8.58583239
C	-9.27196848	5.33426336	-9.19677361
C	-11.29637854	7.18332133	-9.65380458
H	-12.25137250	6.35448783	-7.93363064
C	-9.16570249	6.22697585	-10.25800974
H	-8.47888117	4.60672057	-9.04912395
C	-10.18008188	7.16848293	-10.50954161
H	-12.09436322	7.89828006	-9.82427913
H	-8.29391409	6.19186251	-10.90282868
N	-6.86651175	3.14816256	3.47775697
N	-1.70645159	4.96850919	12.72555860
C	-10.58175012	9.40144058	-11.47043017
C	-10.32652983	10.15823305	-10.31496260
C	-11.33270322	9.97478127	-12.50950182
C	-10.82429174	11.45601967	-10.20047024
H	-9.73820322	9.72608532	-9.51154210
C	-11.81134891	11.27978846	-12.39426088
H	-11.53887026	9.39410746	-13.40304343
C	-11.56540834	12.02681449	-11.23899800
H	-10.61754864	12.02798847	-9.29981648
H	-12.39087600	11.70792667	-13.20770003
C	-9.48566687	7.66348565	-12.82121104
C	-8.57559133	8.50386887	-13.48264899
C	-9.80639464	6.42068388	-13.39132104
C	-8.00482239	8.10818483	-14.69226352
H	-8.31968740	9.46383635	-13.04525394
C	-9.21708407	6.02498001	-14.59193924
H	-10.51786556	5.77069483	-12.89174534
C	-8.31683249	6.86611158	-15.25191153
H	-7.30261937	8.77041788	-15.19152394
H	-9.47648146	5.06037995	-15.01989276
H	-7.86551865	6.55833760	-16.19065335
N	-10.08064831	8.07345255	-11.59274886
H	0.11084304	5.58352278	16.03398947
H	-11.94513471	13.04046420	-11.14963658

^{1}H NMR spectrum of **1**$(PF_6)_4$ in CD_3CN:

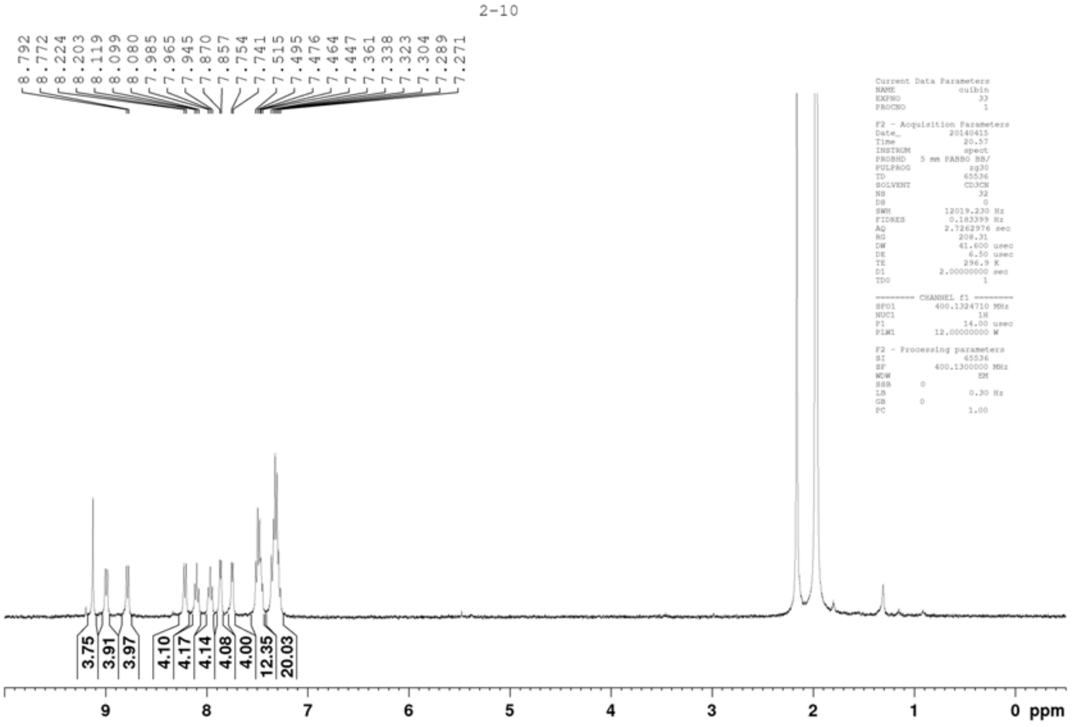

^{13}C NMR spectrum of **1**$(PF_6)_4$ in CD_3CN:

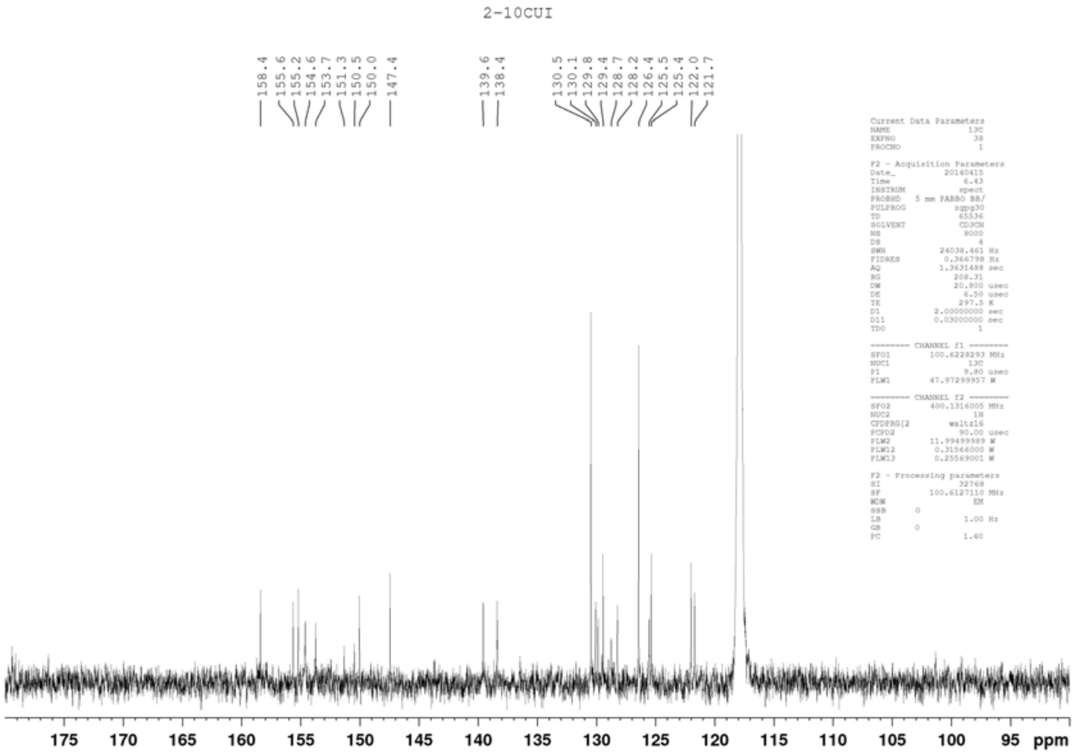

MALDI-TOF mass spectrum of **1**$(PF_6)_4$:

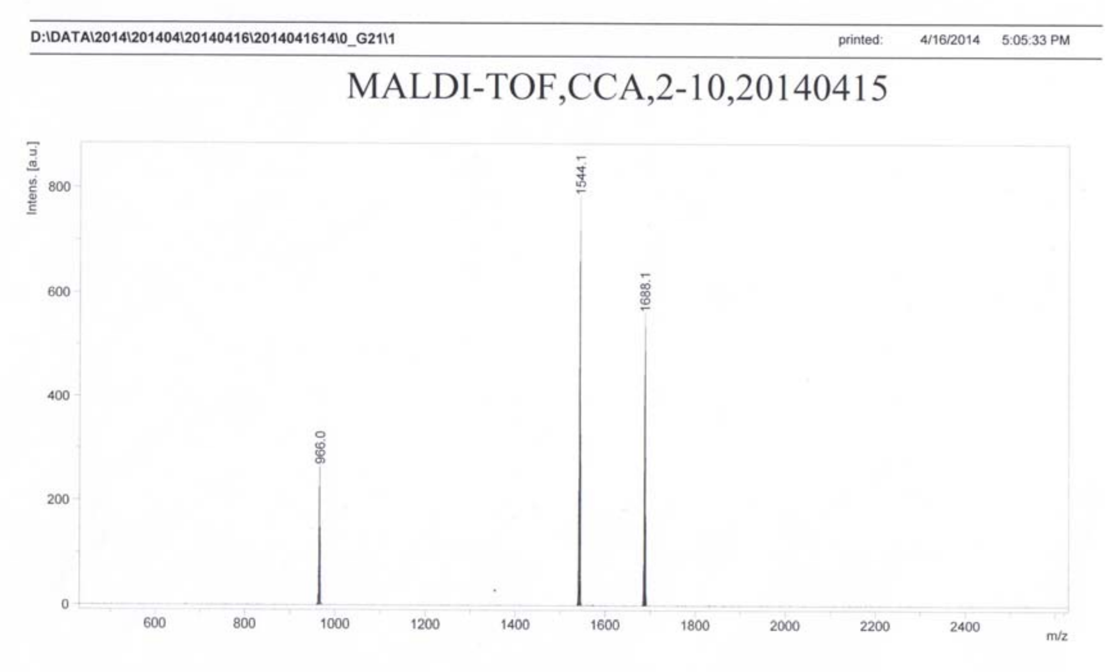

^1H NMR spectrum of **2**$(PF_6)_2$ in CD_3CN:

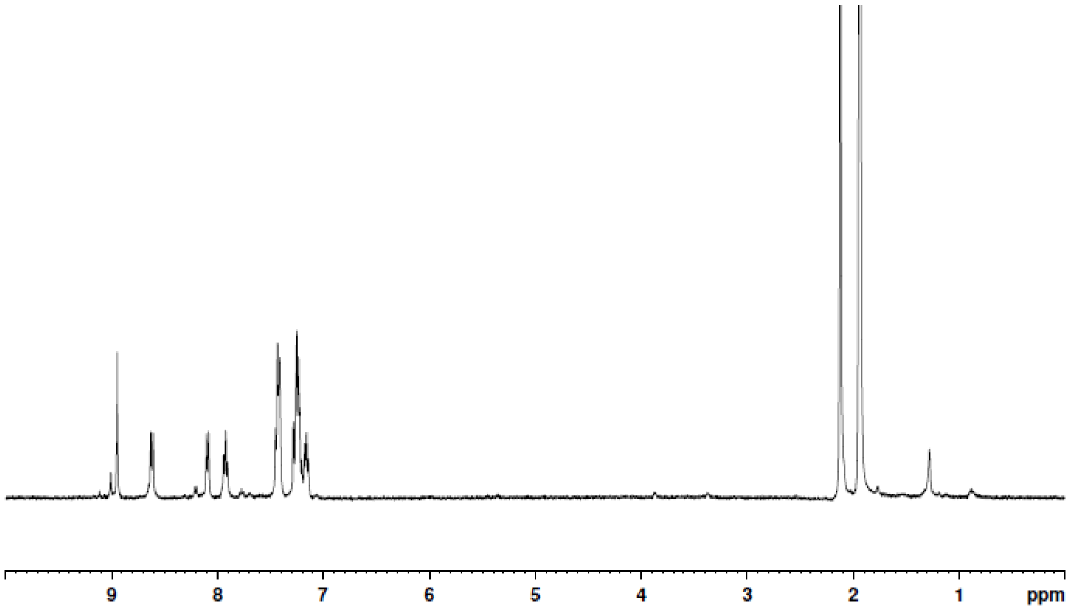

MALDI-TOF mass spectrum of **2**$(PF_6)_2$:

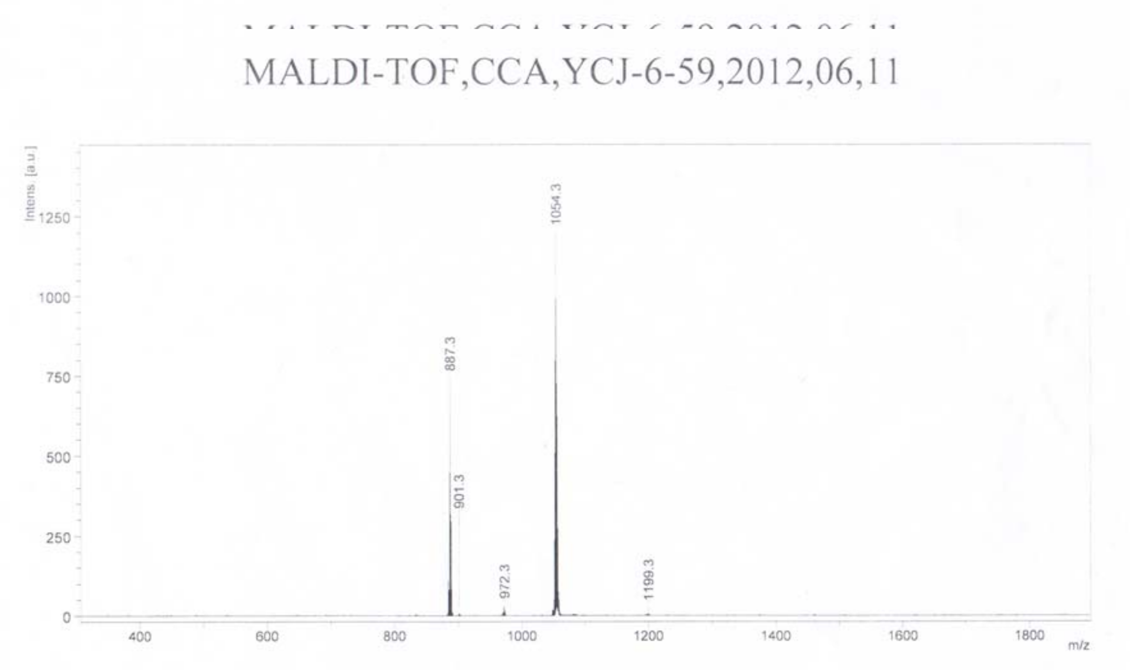

Supplement: Supplementary file 1 [file SC-006-C4SC03345K-s001.pdf]
